# Is patient-centred care for women a priority for policy-makers? Content analysis of government policies

**DOI:** 10.1186/s12961-020-0533-z

**Published:** 2020-02-18

**Authors:** Anna R. Gagliardi, Sheila Dunn, Angel M. Foster, Sherry L. Grace, Nazilla Khanlou, Donna E. Stewart, Sharon E. Straus

**Affiliations:** 1 Toronto General Hospital Research Institute, University Health Network, 200 Elizabeth Street, Toronto, M5G2C4 Canada; 2 Department of Family and Community Medicine, University of Toronto and Women’s College Research Institute, Toronto, M5S1B2 Canada; 3 Faculty of Health Sciences, University of Ottawa, Ottawa, K1N6N5 Canada; 4 York University and University Health Network, Toronto, M3J1P3 Canada; 5 York University, Toronto, M3J1P3 Canada; 6 University of Toronto, Toronto General Hospital Research Institute, Toronto, M5G2C4 Canada; 7 Keenan Research Centre of the Li Ka Shing Knowledge Institute, St. Michael’s Hospital, Toronto, M5B1W8 Canada

**Keywords:** Patient-centred care, women’s health, government policy, evidence-informed policies, qualitative content analysis

## Abstract

**Background:**

Considerable research shows that women experience gendered disparities in healthcare access and quality. Patient-centred care (PCC) could reduce inequities by addressing the patient’s clinical and personal needs. Healthcare policies can influence service delivery to optimise patient outcomes. This study assessed whether and how government policies recognise and promote PCC for women (PCCW).

**Methods:**

We analysed the content of English-language policies published in Canada from 2010 to 2018 on depression and cardiac rehabilitation – conditions featuring known gendered inequities – that were identified on government websites. We extracted data and used summary statistics to enumerate mentions of PCC and women’s health.

**Results:**

We included 30 policies (20 depression, 10 cardiac rehabilitation). Of those, 20 (66.7%) included any content related to PCC (median 1.0, range 0.0 to 5.0), most often exchanging information (14, 46.7%) and making decisions (13, 43.3%). Less frequent domains were enabling self-management (8, 26.7%), addressing emotions (6, 20.0%) and fostering the relationship (4, 13.3%). No policies included content for the domain of managing uncertainty. A higher proportion of cardiac rehabilitation guidelines included PCC content. Among the 30 policies, 7 (23.3%) included content related to at least one women’s health domain (median 0.0, range 0.0 to 3.0). Most frequently included were social determinants of health (4, 13.3%). Fewer policies mentioned any issues to consider for women (2, 28.6%), issues specific to subgroups of women (2, 28.6%) or distinguished care for women from men (2, 28.6%). No policies included mention of abuse or violence, or discrimination or stigma. The policies largely pertained to depression. Despite mention of PCC or women’s health, policies offered brief, vague guidance on how to achieve PCCW; for example, “*Patients value being involved in decision-making*” and “*Women want care that is collaborative, woman- and family-centered, and culturally sensitive*.”

**Conclusions:**

Despite considerable evidence of need and international recommendations, most policies failed to recognise gendered disparities or promote PCC as a mitigating strategy. These identified gaps represent opportunities by which government policies could be developed or strengthened to support PCCW. Future research should investigate complementary strategies such as equipping policy-makers with the evidence and tools required to develop PCCW-informed policies.

## Background

Healthcare policies can widely influence the quality of service delivery and associated patient outcomes; however, policies frequently fail to reflect and address the needs established by healthcare research. For example, analysis of 150 regional health board policies in England found that 52% mentioned end-of-life care but none specified strategies to support it [1]. Analysis of 21 government physical activity policies in 6 European Union countries found that research was used to justify policy creation but did not inform the policies [2]. Common barriers to the use of evidence cited by policy-makers include a lack of evidence relevant to health policy, cost considerations and an absence of shared understanding about priorities between policy-makers and researchers [3]. A scoping review of 22 studies from 18 countries published from 1999 to 2016 found that tailored interactive workshops supported by goal-focused mentoring and that collaboration with researchers can increase policy-makers’ use of research [4].

The quality of care for women has been a long-standing concern, so whether and how recommendations pertaining to gendered disparities are translated into policy and practice warrants evaluation. In 1995, the Fourth World Conference on Women convened by the United Nations revealed the need to deliver services that are sensitive to the needs and preferences of women [5] and, in 2009, the WHO report, Women and Health, emphasised the need to improve the quality of women’s healthcare services [6]. Since then, the concept of women’s health has broadened from a focus on sexual and reproductive health to a life-course approach that considers other health challenges that affect women during and beyond their reproductive years [7]. Yet, considerable research shows that women are less likely than men to have medical needs addressed, access to a specialist, or report good patient–provider communication [8–15]. Such gendered disparities may be heightened by social determinants of health in both developed [16] and developing countries [17], which may influence women’s access to services or their healthcare experience. Hence, promoting women’s health and well-being by ensuring that healthcare is responsive to gender and the life course is one of 17 goals in the United Nations report Gender Equality in the 2030 Agenda for Sustainable Development [18].

Patient-centred care (PCC), originally defined by the Institute of Medicine as healthcare that establishes a partnership among practitioners, patients and their families to ensure that care is attentive to the needs, values and preferences of patients, is one approach to reduce persisting gendered disparities among women because it tailors care to an individual patient’s clinical needs, life circumstances that may reflect social determinants of health and personal preferences [19]. In this way, PCC can overcome potential barriers in access to care at the patient level and improve the healthcare experience. Furthermore, PCC is considered a fundamental element of high-quality healthcare because it has improved patient experience and clinical outcomes for many conditions across settings, including primary, emergency, acute and intensive care [20–22]. Hence, PCC was advocated by the United Nations and WHO to mitigate gendered disparities in healthcare quality [5, 6, 18]. There is no universal shared understanding of PCC. Some frameworks include organisation and system-level determinants of access to care. In keeping with the Institute of Medicine definition of PCC as an approach to patient–provider communication, common elements across more than 25 PCC frameworks suggest that clinicians can achieve PCC by fostering a healing relationship, exchanging information, addressing emotions or concerns, managing uncertainty about treatment risks and benefits, engaging patients in decision-making, and enabling self-management [23, 24]. Recommendations generated by consensus among women, women’s health experts, and health system leaders advocate for public health leadership that promotes women’s health by considering gender and health in all government policies [25, 26]. Given the imperative to improve PCC for women (PCCW) and the potential for government policies to guide, incentivise and support health system change and improvement, we aimed to assess if and how government policies recognise and promote PCCW. If found, exemplar policies could be broadly emulated or, if lacking, this would identify opportunities by which government policies could be strengthened to better promote PCCW.

## Methods

### Approach

This research employed qualitative content analysis of government policies to assess if and how they addressed PCCW [27], an approach commonly used in other policy analyses [1–3]. This approach can be used to qualitatively and/or quantitatively describe explicit content in written, verbal or visual communication without any theoretical interpretation. We employed directed/deductive and summative content analysis techniques to describe how policies identified or promoted PCCW (directed/deductive) and enumerate the number and type of policies that addressed PCCW (summative) [28]. To enhance rigour, we addressed reflexivity in all stages of the research through independent analysis by multiple team members (MZ, DK, BN, JR, TF, ARG) and through independent review and interpretation of the findings by the research team, which included researchers with expertise in the conditions of interest, physicians, Chairs of Women’s Health Research, and representatives of quality improvement and professional organisations. We complied with the Standards for Reporting Qualitative Research [29]. We obtained data from publicly available sources so informed consent was not needed.

### Eligibility

For this study, we included national and provincial policies from Canada. We defined government as a national or regional government, governmental ministry of health, or agency with government-delegated authority over healthcare delivery or monitoring. We defined a policy as a document generated or sponsored by government to guide the planning, organisation, delivery or improvement of healthcare programmes or services. Policies were labelled with a variety of terms, including policy, decision, plan, report, framework or strategy. We used the PICO framework (participants, intervention or issue, comparisons or publication types, outcomes) to inform inclusion criteria. Participants referred to government policies for adults aged 18+ years. Issues referred to policies pertaining to depression or cardiac rehabilitation (or mental or cardiovascular health, more broadly), conditions with known gendered disparities in care and outcomes worldwide [8–15]. For example, compared with men, women with acute myocardial infarction underwent fewer procedures, had significantly higher risk of 30-day readmission, and were less likely to be referred for cardiac rehabilitation; additionally, women reporting depression were less likely to be referred or treated for those symptoms. Policies were also included if they pertained to women’s health in general. Comparisons/publications referred to publicly available government policies published in English language after January 1, 2010. We chose this date to correspond with publication of evidence showing gendered inequities in the conditions of interest [9], and of recommendations for policies to address gender and health [26] in Canada, which may have stimulated the inclusion of PCCW in policies based on raising broad awareness of these issues. Outcomes referred to any goals or objectives specified in the policies for healthcare programmes or services. We excluded policies if they were developed by professional societies or quality improvement agencies, or were clinical guidelines or health technology assessments. The research team reviewed and offered suggestions to refine eligibility criteria.

### Searching and screening

Two research associates (MZ, DK) searched the Internet with Google to compile a list of government websites. On those websites, MZ and DK identified eligible policies by searching with keywords (i.e. cardiovascular, cardiac or depression and policy, report, plan, framework, programme or strategy) and browsing with navigation menu options (i.e. publications, condition of interest). Three research associates (MZ, DK, BN) and ARG independently screened a sample of 15 policy titles/descriptions and discussed their results to refine eligibility criteria and the approach to screening. DK and BN then independently screened remaining policy titles/descriptions, periodically consulting with ARG to address uncertainties or discrepancies.

### Data collection and analysis

We developed a data extraction form to collect information on policy characteristics (year of publication, government, clinical topic, policy objective), and PCC and women’s health content. PCC content referred to any mention of PCC approaches: fostering a healing relationship, exchanging information, addressing emotions, managing uncertainty or prognosis or treatment outcomes, decision-making, and enabling self-management, which are common domains across widely used PCC definitions and frameworks derived from the recommendations of patients and healthcare professionals [23, 24]. Women’s health content referred to any mention of women’s health, gender considerations or determinants of health among women (i.e. education, socioeconomic status). As a pilot test, DK, BN and ARG independently extracted data from three government policy documents, then compared and discussed results to establish a shared understanding of what to extract. DK and BN independently extracted data from remaining policies. ARG, with help from research assistants JR and TF, resolved uncertainties and discrepancies. We used summary statistics to report policy characteristics, and the number and type of policies that addressed PCC and women’s health. We used text to describe how policies addressed PCC and women’s health. The larger research team reviewed summary data and assisted with interpreting study findings.

## Results

### Policy characteristics

We identified a total of 30 eligible policies relevant to the conditions of interest [30–59]. Extracted data are included in Additional file 1. Policies were published between 2010 and 2018. With respect to policy topic, 18 (60.0%) pertained to general health, 11 (36.7%) were specific to either depression or cardiac rehabilitation, and 2 (6.7%) were specific to women’s health. By condition, 20 (66.7%) policies addressed depression, and 10 (33.3%) addressed cardiac rehabilitation. Policy objectives were to report on planned strategies or programmes (17, 56.7%), population health audit (8, 26.7%), or evaluation of strategies or programmes (5, 16.7%). Table 1 summarises PCCW content overall and by condition. Table 2 offers examples of PCCW content, both limited and expanded.

**Table 1:** Patient-centred care for women (PCCW) content in policies overall and by condition

PCCW domains	Policies by condition (*n*, %)		Total (n, %) *n* = 30
	Depression *n* = 20	Cardiac rehabilitation *n* = 10	
Patient-centred care			
Fostering relationship	1 (5.0)	3 (30.0)	4 (13.3)
Exchanging information	7 (35.0)	7 (70.0)	14 (46.7)
Addressing emotions	2 (10.0)	4 (40.0)	6 (20.0)
Managing uncertainty	0 (0.0)	0 (0.0)	0 (0.0)
Making decisions	8 (40.0)	5 (50.0)	13 (43.3)
Enabling self-management	4 (20.0)	4 (40.0)	8 (26.7)
Women’s health			
Issues to consider for women	2 (10.0)	0 (0.0)	2 (28.6)
Social determinants of health	3 (15.0)	1 (10.0)	4 (13.3)
Issues specific to subgroups (i.e. immigrants)	2 (10.0)	0 (0.0)	2 (28.6)
Distinguished care for women from men	2 (10.0)	0 (0.0)	2 (28.6)
Abuse or violence	0 (0.0)	0 (0.0)	0 (0.0)
Discrimination or stigma	0 (0.0)	0 (0.0)	0 (0.0)

**Table 2:** Examples of patient-centred care for women (PCCW) content in include policies

PCCW domains	Recognition or guidance of domain	
	Limited	Expanded
Patient-centred care	“*Provide patient and family-centred care that supports the whole person and enables optimal self-management*” [53]	“*Effective self-management is very different from telling patients what to do. Patients have a central role in determining their care – one that fosters a sense of responsibility for their health. Use effective self-management support strategies that include assessment, goal setting, and action planning, problem-solving and follow-up. Organise internal and community resources to provide ongoing self-management support to patients. Patients develop personal skills to maintain their health and wellness*” ([59], p. 16). “*Have modules on goal setting, action planning, managing challenges in chronic disease, etc*.” ([59], p. 16). Also discusses the need for providers to follow-up with patients and provide follow-up reminders [59].
Women’s health	“*Women want care that is collaborative, woman- and family-centered, and culturally sensitive*” [42]	“*Women and men are affected by different health issues and often have differing health-care needs. For example, they have different morbidity and mortality patterns as well as differing experiences with healthcare. However, differential health outcomes are not only linked to biology (sex), but to overall life circumstances and experiences of women and men based on gender, among other diversity factors. For health issues common to women and men, equity does not necessarily mean the provision of the same treatment, but rather the provision of treatment that is fair and which will result in equality of outcomes*.” ([38], p. 20)

### Patient-centred care

Among the 30 policies, 20 (66.7%) included content related to at least one PCC domain (median 1.0, range 0.0 to 5.0) (Additional file 2). In those 20 policies, the most frequently included domains were exchanging information (14, 46.7%) and making decisions (13, 43.3%). Less frequent domains were enabling self-management (8, 26.7%), addressing emotions (6, 20.0%) and fostering the relationship (4, 13.3%). No policies included content for the domain of managing uncertainty. A higher proportion of cardiac rehabilitation guidelines included PCC content.

However, even when policies included content related to PCC domains, those descriptions were often brief and vague, offering little to no concrete guidance on what should be done to achieve PCC. For example, one policy recommended to “*provide patient and family-centred care that supports the whole person and enables optimal self-management*” [53]. In contrast, a more informative policy emphasised engaging patients to foster a sense of responsibility, using support strategies that include assessment, action planning, goal setting or problem solving, offering access to community support services, and following up with reminders [59].

### Women’s health

Among the 30 policies, 7 (23.3%) included content related to at least one women’s health domain (median 0.0, range 0.0 to 3.0) and 6 (20.0%) included both PCC and women’s health (Additional file 3). Of the 7 policies that included any women’s health domains, most frequently included were the social determinants of health (4, 13.3%). Fewer policies mentioned any issues to consider for women (2, 28.6%), issues specific to subgroups of women (2, 28.6%) or distinguished care for women from men (2, 28.6%). No policies included mention of abuse or violence, or discrimination or stigma. These largely pertained to policies on depression because only one policy on cardiac rehabilitation addressed women’s health by mentioning the social determinants of health [49].

Despite mention of these factors, no policy offered concrete guidance on how to improve women’s health or gendered disparities. For example, one policy noted that “*Women want care that is collaborative, woman- and family-centered, and culturally sensitive*” but did not explain how that could be achieved [42]. In contrast, a more informative policy recognised that men and women have different morbidity and mortality patterns, and different health experiences and associated outcomes based on gender, recommending that treatment should be fair [38].

## Discussion

Among 30 government policies published from 2010 to 2018 on depression or cardiac rehabilitation – conditions with known gendered inequities in access to and quality of care – many included content relevant to at least one PCC domain considered important by patients and healthcare professionals but offered no concrete guidance on how to achieve PCC. Additionally, few mentioned women’s health or included content about healthcare disparities or guidance on how to address them. Thus, despite considerable evidence of need and international recommendations, policies fail to recognise or promote PCCW.

Few studies prior to this one had examined policies for guidance on gendered inequities in healthcare. In the United States, 51.0% of 77 public health policies at the state, organisational or school level were assessed as being gender aware [60]. A narrative synthesis of literature found that policy reforms or strategies achieved limited success in improving gender equality in health [61]. Four European case studies showed that policies supporting women’s participation in the labour force or decreasing their burden of care reduced gendered inequalities in health status [62, 63]. These studies are relevant but focus on social and public health strategies rather than health services. Our study was unique in that it specifically examined whether and how government health policies incentivised, promoted or guided the planning, delivery, evaluation or improvement of healthcare services to optimise PCC or women’s health. Our findings are consistent with the above-cited work that identified deficiencies in how policies address gendered inequities.

The finding that government health policies do not recognise, emphasise, prioritise or promote PCCW raises two issues with implications for ongoing research. First, we and others [1–3] have demonstrated a need to promote the development of evidence-informed policies. Synthesised research showed that interactive workshops or collaboration with researchers can improve the use of research by policy-makers [4]. However, analysis of 131 policies and interviews with policy-makers that developed them found that access to and relationships with researchers increased the interaction between policy-makers and researchers but did not lead to the use of research in policy-making [64]. Hence, more research is needed to understand how to make policies more responsive to the healthcare needs that are clearly demonstrated through research. One option is to equip policy-makers with tools by which they can develop evidence-informed policies. For example, Gavriilidis et al. [65] developed an evaluation framework by which to assess if policies promote gender equity and health. Another option is to equip healthcare professionals with tools by which they can adapt or transform policies into evidence-informed healthcare services. For example, Themessl-Huber et al. [66] generated a framework to evaluate whether programmes are responsive to local contexts and stakeholders. More recently, Canada introduced an action plan at the federal level to engage in evidence-based policy and programme design informed by sex, gender and diversity research [67], and implemented requirements for investigators to address sex and gender in proposals submitted to research funding competitions [68]. These initiatives may incentivise future policies to address PCCW.

Second, in prior research, policy-makers have said that the lack of relevant research is a barrier to developing evidence-informed policies. While considerable evidence has accumulated on gendered disparities in healthcare [8–18], less is known about what constitutes PCCW. We synthesised research published from 2008 to 2018 that investigated PCC and included at least 50% women and identified only four studies on depression and one on cardiovascular rehabilitation [69]. More research is needed to fully conceptualise and describe PCCW across different conditions, and to test interventions that improve PCCW. Sharing that evidence with policy-makers could help them to generate policies that address PCCW.

The strengths of our study include the use of rigorous methods for content analysis [27, 28] that involved independent searching, screening and data extraction by multiple individuals, compliance with standards for the reporting of qualitative research [29], and the use of an established PCC framework upon which to map policy content [24]. Several factors may limit the interpretation and application of the findings. The search strategy we employed may not have identified all relevant government policies, plus our search was restricted to English-language policies and did not include French-language policies from the province of Quebec. Furthermore, our review included policies that addressed two clinical topics only, thus it is not known if the findings are transferrable to policies on other clinical topics. There is no universal agreement on what constitutes PCC, thus the PCC framework we employed is not necessarily a gold standard [24], and what constitutes PCC may differ by condition. The PCC framework pertains to patient–clinician interaction and does not necessarily address system-level issues that policies typically address.

## Conclusions

Analysis of 30 government healthcare policies from Canada on depression or cardiac rehabilitation revealed that gendered disparities in quality of care remained largely ignored in general, and revealed little recognition of PCC or guidance on how PCC can mitigate gendered disparities in care or improve women’s healthcare experiences more specifically. Despite considerable evidence of gendered inequities in access to and quality of care for depression or cardiac rehabilitation, PCCW has not been prioritised by policy-makers. These identified gaps represent opportunities by which government policies could be developed or strengthened to support PCCW. Other strategies include equipping policy-makers or healthcare professionals with the tools to develop PCCW-informed policies or to adapt policies into PCCW-tailored healthcare services.

## Supplementary information

**Additional file 1.** Data extracted from included government policies. Details that describe policy characteristics and content.

**Additional file 2.** Patient-centred care domains addressed in included policies. Checklist of domains included in each policy.

**Additional file 3.** Women’s health domains addressed in included policies. Checklist of domains included in each policy.

## Data Availability

All data generated or analysed during this study are included in this published article and its supplementary information files.

## Abbreviations

PCC: patient-centred care
PCCW: patient-centred care for women
