# Supplementary material for: Is patient-centred care for women a priority for policy-makers? Content analysis of government policies
Source: Health Res Policy Syst. 2020 Feb 18;18:23. doi: 10.1186/s12961-020-0533-z (PMC7029558; doi:10.1186/s12961-020-0533-z)
Supplement: Supplementary file 1 — Additional file 1. Data extracted from included government policies. Details that describe policy characteristics and content. [file 12961_2020_533_MOESM1_ESM.docx]

**Additional File 1. Data extracted from included government policies**

**Depression (or mental health)**

| Policy  Title [reference]  Country  Date | Objective | Patient-centred care | Women’s health |
| --- | --- | --- | --- |
| Ontario Ministry of Health and Long- Term Care Estimates Briefing Book 2017-18 [30]  2018 | The estimates briefing book aims to outline the Ontario Ministry of Health and Long Term Care planned expenditures, priorities and results of Ministry programs from 2017-2018. | **Decision-Making**  -The Patient and Family Advisory Council will help ensure: (p14)  -Patients, caregivers, and families have a stronger voice in health care policy decision-making. | The ministry is supporting the establishment of six Indigenous Midwifery programs across the province to provide culturally appropriate child and maternity care to Indigenous women, their children and families. Indigenous midwives will provide a full range of safe and culturally appropriate midwifery primary care, support services, language and community education to pregnant women and the broader community. (P.8) |
| The Status of  Women in Manitoba 2018 [31]  2018 | This report is intended to be used as a valuable source of gender-disaggregated data that will help all Manitobans explore issues and trends related to gender equality in our province. Governments, civil society, the private sector and institutions can use this information to advance gender equity in Manitoba. We hope the publication will aid the continuing discussion and  evaluation of the changing roles and social characteristics of women in Manitoba, as well as contribute to the development of policies improving the status of women in our province. (p2) | **---** | --- |
| Mental Health  and Addictions  Realizing  the Vision  Better Mental Health Means Better Health [32]  2017 | The third and final Report of Ontario’s Mental Health & Addictions Leadership Advisory Council on the government’s 10-year strategy to build a comprehensive mental health and addictions system in Ontario. The purpose is to provide a final set of recommendation for this strategy | **Exchanging information:**  Enhance mental health literacy among Ontarians through public awareness initiatives that emphasize universal wellness for all, with differentiated knowledge-building for those who are particularly well-positioned to identify and support individuals with mental health problems (such as early childhood educators, primary care physicians and nurse practitioners, school staff, coaches, campus staff,  faith leaders, and parents/guardians). (p5)  **Patient Self Management:**  creating training opportunities for future and current members of the mental health and addictions workforce to learn from people with lived experience about their experiences of the system, as well as of wellness and recovery; increasing the number of mental health and addictions peer support workers across Ontario  in a range of settings; p12 | --- |
| Milestones on Our Journey  Transforming Mental Health and Addictions in Nova Scotia  A Provincial Model for Promoting Positive Mental Health, Care and Support [33]  2017 | The purpose of this report is to document the evolution of our work and to identify priorities for change that align with national strategies to transform mental health and addiction care and support across Canada. (p4)  The overall goal for the Advisory Committee was to strategically plan for a full continuum of services and systems supports to better meet the needs of Nova Scotians across their lifespans. The plan is intended to serve as a road map or framework, which will help further guide the development and implementation of a provincial model for Promoting Positive Mental Health, Care and Support in Nova Scotia.  The goal of this report, titled Milestones on Our Journey to Transform Mental Health and Addictions in Nova Scotia (the Milestones Report), is to share our experiences and highlight the key milestones in our planning journey to date (p9) | **Decision-making**  Improve the meaningful involvement of clients/patients and families in clinical decision making as well as in planning and evaluating initiatives designed to improve MHA. (P.55)  **Exchanging information**  The need to incorporate the perspective of patients, clients and their families in the planning process if we are to truly achieve patient-centred care as an outcome; (p28) ensuring people’s presenting needs are appropriately understood and matched with the right intensity or level of service/care. It is important people and their families are not being over treated or underserviced. (p47) Increase the use of technology and e-mental health solutions to foster collaboration, support, education, increased access to consultative and other services, and engage people and their families. (p55) | --- |
| Transformational Roadmap  Addiction & Mental Health Strategic Clinical Network [34]  2017 | To improve addiction and mental health patient care and health outcomes in Alberta by engaging stakeholders to identify meaningful, evidence based opportunities for transformational change. (p4) | **---** | --- |
| Towards Recovery: The Mental Health and Addictions  Action Plan for Newfoundland and Labrador [35]  2017 | The goal of this plan is to guide the implementation of the recommendations outlined in Towards Recovery, and  provide direction for mental health and addictions policy and programs for the next five years (2017 to 2022) | **Self-Management**  Encourage community leaders to form coalitions to promote mental wellness, encourage people to seek help and prevent suicide (p14) Provide web-based information on education, self-care and self-management for families and caregivers (p18) Through a stepped-care approach, develop and offer a range of mental health and addictions services integrated, wherever possible, within existing community and primary health care services throughout the province, including:- Self-management programs for mental wellness, anxiety and depression (p21)  **Exchanging Information**  Provide online information about the mental health and addictions services and how to navigate them. Ensure primary health care providers have improved access to information about programs and services to share with families.  **Making Decisions**  Develop standards and guidelines, which recognize the critical role of families and caregivers, and provide guidance to staff on how to support families and caregivers, and include them, wherever possible, in treatment decisions. | --- |
| Provincial Clinical and Preventative Services Planning for Manitoba: Doing things differently and better [36]  2017 | This report is a continuum of the environmental scan submitted on December 1, 2016, and is, effectively, a critical narrative built around an evidence-based nucleus of forecasting tables. Rather than a roadmap, it is the beginning of an ongoing exercise in the province, one which will never end.  The environmental scan addressed details of the critical roles of non-physician providers of care and the need to expand those roles to the full capacity and knowledge of individual providers and groups of providers. (p5) | **Responding to and addressing emotions**  Traumatizing events can take a serious emotional toll on those involved, even if the event did not cause physical damage. This can have a profound impact on the individual’s identity, resulting negative effects in mind, body, soul, and spirit.10 It is stressed that trauma is determined, not by the triggering event(s), but the individual’s experience and its meaning to the individual. (p49)  **Fostering healing relationships**  This requires an understanding that physicians place high value on their clinical autonomy and professional self-determination; hence, the importance of building relationships and gaining trust through  consultation and dialogue in order to achieve the sense of collective responsibility for quality improvement. (p122)  **Enabling patient self-management**  expansion of local expertise and support to enable the majority of patients to be managed close to home, reserving the transportation to Winnipeg or Brandon for select complex cases. This overall approach was considered best to align with the objectives of quality, accessibility, and patient-centred. (p89) PACT is an outreach oriented comprehensive community treatment, rehabilitation, and support service designed to meet the needs of people with severe and persistent mental illness. The service is provided to participants in their homes, at work, and in community settings. (p91)  The Crisis Response Centre (CRC), located at HSC, is a 24/7 service that provides walk-in assessment and treatment for those in mental crisis; CRC also provides referrals to other mental health services. (p93) | --- |
| Moving Forward Together, Prince Edward Island’s Mental Health and Addictions Strategy, covering the period 2016 to 2026 [37]  2016 | offers a comprehensive approach to transforming the mental health system through a clear vision, interconnected strategic priorities and long-term strategies for change.  This strategy identifies some of the challenges the system faces; describes anticipate outcomes; and identifies five strategic priorities that will be the focus of the next 10 years | **Decision Making:**  -empowered people and families to make choices and decisions about their own mental health care  -People who experience, or are at risk of experiencing, mental health and substance abuse problems must be partners in their care (p1) | --- |
| Health Inequities in New Brunswick: A Report from the Office of the  Chief Medical Officer of Health [38]  2016 | This report is meant to stimulate individual and  collective interest and facilitate conversations to address this important issue which affects all New Brunswickers one way or another. The report illustrates some of the differences in health experiences of New Brunswickers based on  household income quintile, showing a clear link between wealth and health; based on where people live, knowing that different parts of the province have different levels of social advantage; based  on education, knowing that opportunities for health increases with education; and based on sex, knowing that women and men have different patterns of illness, risk factors, risk behaviours and health experiences. (p5) | --- | Women and men are affected by different health issues and often have differing health-care needs. For example, they have different morbidity and mortality patterns as well as differing experiences with health care. However, differential health outcomes are not only linked to biology (sex), but to overall life circumstances and experiences of women and men based on gender, among other diversity factors. For health issues common to women and men, equity does not necessarily mean the provision of the same treatment, but rather the provision of treatment that is fair and which will result in equality of outcomes. (p20) |
| Health PEI  Annual Report  2016-2017 [39]  2016 | Working in partnership with Islanders to support and promote health through the delivery of safe and quality  health care. (p3) | **Patient self-management**  Expanded home care services to include an additional 30 full-time equivalent (FTE) social workers to provide care coordination and adult protection services. (p24) Thirty staff in CMH received training in Dialectical Behaviour Therapy which supports suicide prevention and trauma support groups. (p16) | --- |
| Provincial Advisory Council  Addiction & Mental Health  2015-16 Annual Report [40]  2016 | The council, in partnership with Alberta Health Services’ provincial addiction and mental health service providers, offers recommendations on addiction and mental health service delivery and program design. The council serves as a conduit for addiction and mental health issues and concerns from communities across Alberta. We make recommendations to Alberta Health Services about programs that need to be developed and we review existing addiction and mental health programs for system improvement. (p6) | **---** | --- |
| Enabling Effective, Quality Population and Patient-centred Care: A Provincial Strategy for Health Human Resources. [41]  2015 | This policy paper sets out both a framework and direction for health human resources in B.C. The proposed framework is designed to structure and align our actions across different levels (practice, regional/organizational, and provincial), across the scope of service delivery (public health, community, diagnostics and pharmacy, and hospital), and across delivery settings (metro, urban, rural, remote). The policy paper is also proposing a number of specific key actions to implement a comprehensive health human resources (HHRM) strategy that will drive the change required to achieve quality services and health outcomes for British Columbians over the coming decade, starting in 2015. | **---** | --- |
| Primary and Community Care in BC: A Strategic Policy Framework [42]  2015 | The goals and objectives of this policy paper align with the strategic direction for the health system in Setting Priorities for the BC Health System (Priorities 1, 2, 3, 4, 5, 7 and 8) and the areas of focus set out in the BC Health System Strategy Implementation: A Collaborative and Focused Approach published in April 2014 by the Ministry of Health  This policy paper aims to both focus and reenergize the commitment to achieve the 2017 vision of the Charter. It also builds on the work and learnings from the past twelve years, the work currently underway and the themes, and a fresh look at the systems current capacity against changing population and patient needs. (p1) | **Decision-making**  Communication and patient-centred care: Patients value being involved in decision-making; receiving accurate information, education, coordination of care, respect for preferences, (P.63)  To ensure access to a comprehensive range of appropriate services for the population they serve, they need to assist patients with healthcare decision-making and assist them to access other levels of the healthcare system, community resources and social services. (P.121)  **Patient self-management**  Parenting programs that aim to improve psychosocial health of parents to help reduce depression, anxiety or stress, and improve self-esteem and relationships. (p63). Telephone based programs like Bounce Back, which is an evidence-based program designed to help adults experiencing symptoms of mild to moderate depression, low mood, or stress, with or without anxiety; (p63). The Rapid Access to Psychiatry initiative is increasing capacity and using existing resources for psychiatric patient care in BC through an alternate model of care that includes group medical visits and/or physician-patient e-mail communication for treatment of patients with depression and anxiety (p92) | Survey data suggests that:  -Pregnant women want to be involved in their care and more specifically in making decisions about their care based upon clinical evidence and the patient’s preferences and values.22  -Women want the use of mobile devices to provide access to health information and services, including the use of self-tracking and other interactive tools that enable pregnant women to receive information (education) individualized to their stage of pregnancy and personalized to their needs.  -Women want care that is collaborative, woman- and family-centered, and culturally sensitive (p35) |
| Progress Report  The Action Plan for Mental Health in New Brunswick  2011–2018 [43]  2015 | This progress report, which builds on the 2013 progress report, highlights the implementation status of the action plan | **Making-decisions:**  The recovery model is not a treatment imposed on an individual. Rather, the role of the health-care system will be to create an environment where recovery can occur. There is a meaningful engagement and partnership; the individual is supported in making informed choices; and goals are set by the individual for improved quality of life. (p4)  **Exchanging information:**  With the placement of the person at the centre of care, success will depend on all stakeholders having an adequate knowledge of current issues in mental health and mental illness. Knowledge enhancement must be accomplished in two fronts. Firstly, persons living with mental illness, as well as their families and other supports must be informed to make educated choices in their treatment plans. Secondly, all health-care and other service providers must be attuned to mental-health issues. (p9) | --- |
| Healthy Environments,  Healthy People  2015 Health Status of Manitobans Report [44]  2015 | This report has been written as a resource for all Manitobans.  The aim of this report is to inspire and stimulate thinking and public discussion on how to move towards a Manitoba where more people have greater opportunities to be  healthy. The report provides an overall assessment on the current health status of Manitobans, and highlights challenges and opportunities in improving the health  and well-being of Manitobans. (p6) | **Exchanging information:** Discusses programs that provide knowledge and information for youth regarding pregnancy (pg. 54), discusses how beliefs are influenced by cultural and religious values (pg. 57).  **Making decisions:** Women are more likely to decide to breastfeed if they have the support of a partner (pg. 43). | Discussed pregnancy as a risk factor for developing mental health issues (pg. 43), discussed women with high risk factors for maternal mental health issues such as poverty, stress, family violence or abuse, pregnancy/ delivery complications, previous history of depression or low social support (pg. 43) |
| New Brunswick’s Wellness Strategy 2014-2021 The heart of our future [45]  2014 | New Brunswick’s Wellness Strategy 2014-2021 is a revised framework and a road map.  The renewed Wellness Strategy supports action on all dimensions of wellness and on all determinants of health. It recognizes and enables the contributions, strengths and skills of New Brunswickers. It can facilitate collective and strategic action, and be a catalyst for dialogue and movement among New Brunswickers. (p5) | **Making Decisions:**  Autonomy refers to the need to have a choice, express ideas and opinions (a voice), and have the ability to make decisions about things that are important to you. When this need is satisfied, in conjunction with other need areas, people tend to make choices that demonstrate respect for themselves and the people around them. | --- |
| Healthy Minds,  Healthy People  A Ten-Year Plan to Address Mental Health  and Substance Use in British Columbia. Monitoring Progress: 2012 Annual Report [46]  2012 | This second annual progress report profiles activities and achievements in the last year through the work  of government, health authorities, non-government organizations, local communities, and business. In  addition, the report highlights how these developments are transforming our approach to mental health and substance use in British Columbia. | **Self-Management**  Action: Improve reach of low-intensity programming, including self-management and supported self-management tools such as the Dealing with Depression program for youth; Strongest Families for children, youth and parents; and the Bounce Back program for adults. (p25). The Bounce Back program, funded by the Ministry of Health and delivered by Canadian Mental Health Association branches throughout the province, offers telephone coaching in a structured self-help program for adults affected by low mood. A self-help DVD is also available. (p26)  In 2012, Fraser Health offered wellness recovery action plan (WRAP) classes throughout the region. WRAP guides participants through the process of identifying and understanding their personal wellness  tools and resources, and helps them to develop individualized plans to use these tools in their day-to-day lives to manage their mental illness. The classes instruct participants in the key concepts of recovery (hope, personal responsibility, education, self-advocacy, and support). WRAP also assists participants to create  advance directives that guide the involvement of family members or supporters when appropriate action on their own behalf is no longer possible and supports participants to develop individualized post-crisis plans for return to wellness. (p31)  **Responding to Emotions**  The B.C. Practice Support program offers focused training  sessions for physicians. Two training modules have been  developed to build capacity to address common mental health problems in primary care, resulting in a reduced reliance on specialized mental health services. The adult mental health module provides family doctors with tools to screen and treat patients for depression and other mental conditions based on cognitive behavioural therapy (p22) | --- |
| Provincial Mental Health and Addictions Advisory Council Activity Plan 2011-2014 [47]  2011 | The Provincial Mental Health and Addictions Advisory Council supports the Department  of Health and Community Services’ Strategic Plan 2011-2017 mission: By March 31, 2017 the Department of Health and Community Services will have provided leadership to support an enhanced health care system that effectively  serves the people of the province and helps them achieve optimal health and  well-being. (p7) | --- | --- |
| Manitoba Women’s  Health Strategy 2011 [48]  2011 | This new Women’s Health Strategy is the result of extensive consultation with  Manitoba women and reaffirms our commitment to providing the best and most appropriate health services to them. The strategy endorses both a vision for women’s health and tangible actions to achieve that vision. The new strategy calls on us to continue to work towards further collaboration and coordination  and assure women take on leadership roles in the health care system. (p1) It seeks to address the broad range of women’s health issues, and  ensure awareness and responsiveness to gender differences in all aspects of  health care delivery in Manitoba (p2) | **Exchanging information:**  Knowledge of women’s health needs must encompass physical, emotional and mental health. This information must be widely shared and available to the public. Share knowledge about women’s health risks, prevention and positive health practices for women.  -Increase understanding about how women’s health issues are different than men’s.  -Increase knowledge about women’s health specific issues amongst girls in public schools.  -Improve access to plain language health information for women, including accessible formats for women with disabilities and older women.  -Increase knowledge and awareness for aboriginal and immigrant women about health issues of concern for them and their families.  **Self Management**  Enable women to gather, support each other and build community through networks and community spaces.  -Develop programs and places that reduce isolation especially for new immigrant women, older women, women with disabilities and for women from northern communities, including those travelling to access services in urban areas.  -Create physical activity programs, information and environments for women and girls. (p8)  Support single parents (mainly women) who are on income assistance and experiencing mental health issues to improve parenting skills, achieve higher marks in school and find employment through the Parent Wellness Initiative. (p10) | The Manitoba Women’s Health Strategy recognizes the distinct health needs of women in the planning, implementation and delivery of health care services. Manitoba’s women are diverse. Gender, race, ethnicity and culture, disability, age, income, geography and sexual orientation have an impact on women’s health status. All Manitoba women need access to health services that take this diversity into account. (p3)  Manitoba’s health system needs to be responsive and sensitive to sex and gender differences and to women’s health needs. Gender-based analysis of all health policies and programs must be part of all health planning and service delivery. (p4) It seeks to address the broad range of women’s health issues, and ensure awareness and responsiveness to gender differences in all aspects of health care delivery in Manitoba. Women make up 51 per cent of the Canadian population. They are frequently the guardians of the health of other family members, and make up the largest number of health care providers and informal caregivers. women face an increased rate of chronic illnesses as they age, as well as higher rates of mental health concerns, such as depression. Issues of violence, poor economic status and lower incomes also affect more women than men.(p2). Gender, race, ethnicity and culture, disability, age, income, geography and sexual orientation have an impact on women’s health status. All Manitoba women need access to health services that take this diversity into account. Gender-based analysis of all health policies and programs must be part of all health planning and service delivery. (P.3) To ensure women’s health needs are reflected, the representation of women in decision-making roles is vital. Women, including Aboriginal, newcomer, women with disabilities, and northern women, must have an effective mechanism to influence government and the health care system by providing input on policies and services that impact their lives. Expanding women-centred health care services including:  -Acute care services for women;  -Health coverage for infertility treatment, and complementary health services;  -Women-specific addiction treatment services including child care  supports and gender specific harm reduction approaches;  -Primary care;  -Mental health services;  -Increased coordination, options and supports for prenatal, birthing and postnatal services and supports across the province,  especially in rural and northern regions;  -Coordinated health information to improve women’s access to personal health information and increase the number of health professionals who are working from the same health information.  **-**Improve supports for specific populations of women including:  -Women with disabilities;  -Lesbian, bi-sexual, trans-gender, two-spirited;  -New Immigrant;  -Sexually exploited youth;  -Older women;  -Aboriginal women, Métis and Inuit women;  -Young girls and teens.  Support single parents (mainly women) who are on income assistance and experiencing mental health issues to improve parenting skills, achieve higher marks in school and find employment through the  Parent Wellness Initiative. (P.10) |
| Report on the Health Status of Manitobans 2010  Priorities for Prevention:  Everyone, Every Place, Every Day [49]  2011 | To report on the health status of Manitobans and assess health-related trends over time. | **Exchange information:** Discuss how cultural beliefs can influence how patients and healthcare providers understand illness (pg. 59).  **Responding to emotions:** Discuss resources that can be used to address emotions, including social supports, regular exercise, avoiding smoking and maintaining a healthy weight (pg. 59).  **Making decisions:** Discuss women’s decision to breastfeed and factors that may influence it such as personal beliefs, supports and health of mother (pg. 80). Discuss comfort and confidence levels of decisions to abstain from substances and how these decisions can lead to **emotional** distress (ex. anxiety, ambivalence) or **uncertainty** (pg. 198). | Factors effecting likelihood to breastfeed included living in higher income neighbourhoods (pg. 80) and factors for problematic pregnancy outcomes include aboriginal women and women living in low income neighbourhoods (pg. 81). |

**Cardiac rehabilitation (cardiovascular health)**

| Policy | Objective | Patient-centred care | Women’s health |
| --- | --- | --- | --- |
| Report from the Canadian Chronic Disease Surveillance System: Heart Disease in Canada [50]  2018 | To provide a national picture of the status of heart disease in Canada and highlight trends from 2000-2013 (pg.5) | **---** | --- |
| Department of Health and Community Services’ 2017-2020 Strategic Plan [51]  2017 | This plan highlights the priorities of the Department for the next three years which are intended to drive continuous improvement in health care and strengthen health outcomes. | **Exchanging information:** Using Telehealth for medical consultation among physicians to assist with patient management in rural communities (pg. 15). Discuss sharing of relevant resources/services to patients (pg. 16). | --- |
| Accreditation Report [52]  2016 | To assess Alberta Health Services' leadership, governance, clinical programs and services against Accreditation Canada requirements for quality and safety as part of the Qmentum Accreditation Program. | **Exchanging information:** Mentions that some teams are in the early stages of exploring how to hear the voices of patients and families (pg. 17). Mentions the sharing relevant information during care (pg. 20).  **Making decisions:** discusses working in partnership with clients and families to ensure they receive the service or procedure intended for them (pg. 20). Information from evaluations should be used to improve transition planning with client and family input (pg. 28). States that guidelines and protocols should include input from clients and families and be regularly updated with this information (pg. 29). Mentions that services should be co-designed with clients and families (pg. 28). Encourages engaging patients and families in care and decision making (pg. 65)  **Fostering healing relationships:** Openness and transparency with clients and families is a strength of these programs (pg. 30).  **Responding to emotions:** Addiction and mental health assessment is done routinely upon admission (pg. 58). | --- |
| A Vision for Chronic Condition and Disease Prevention and Management [53]  2016 | To implement the vision and mission of keeping individuals, families and communities as healthy as they can be through an integrated approach to chronic condition and disease prevention and management (pg. 4) | **Exchanging information:** Integrate information systems that support current information on supports and services available to individuals and families related to chronic disease prevention and management (pg. 5).  **Fostering healing relationship:** Reach out to individuals through other avenues than face to face visit where relationship is established; being proactive rather than waiting for the individual to reach out to us as providers (pg. 12).  **Responding to emotions:** Discuss the role of family, friends and healthcare providers in providing patients with emotional support (pg. 21).  **Making decisions:** Support all providers to engage individuals and families and their supports (as defined by them) in their own care and decision making by providing the training and tools required to do this well (pg. 13).  **Enabling self-management:** Discuss coordinated care developed in partnership with health provider, provide patient and family-centred care that supports the whole person and enables optimal self-management, apply behaviour change principles to support individual and families to better manage health, facilitate patient access to reliable evidence informed information (applying the principles of health literacy) and their personal health record (pg. 5). | --- |
| Health Annual Report 2016–2017 [54]  2016 | Health Canada is the federal department responsible for helping people of Canada maintain and improve their health. On an annual basis, the federal Minister of Health is required to report to Parliament on the administration and operation of the Canada Health Act, as set out in section 23 of the Act. The vehicle for so doing is the Canada Health Act Annual Report. While the principal and intended audience for the annual report is Parliamentarians, it is a public document that offers a comprehensive description of insured health services in each of the provinces and territories. The Annual Report is structured to address the mandated reporting requirements of the Act; as such, its scope does not extend to commenting on the status of the Canadian health care system as a whole. | **---** | --- |
| Setting Priorities for the B.C. Health System [55]  2014 | The four goals of this document were (pg.1):  -Provide effective health promotion, prevention and self-management to improve the health and wellness of B.C. citizens  -Meeting the majority of health needs with high quality healthcare and support services  -Ensuring high quality hospital care services are available when needed  -Improving innovation, productivity and efficiency in the delivery of health services | **Exchanging information:** Implementation of e-Health to make health care information accessible (pg. 18).  **Responding to emotions:** Mentions that mental health needs of populations should be used to understand patients (pg. 9).  **Making decisions:** Discusses implementation of e-health to support personal health and health care decision-making (pg. 18). Improving patient-centered choice and timeliness of access to treatment through the wider use of patient-focused funding programs that may also support different models of providing medical and surgical care, including increased contracting of services out of hospital settings where appropriate (p. 30).  **Enabling self-management:** a key goal of the document is to provide “self-management to improve the health and wellness of British Columbians” (pg. 1). | --- |
| The Provincial Health Plan  2013-2018 [56]  2013 | The provincial health plan provides New Brunswick’s health partners and stakeholders with a  blueprint for the delivery of health services. It is used to manage existing health-care services and resources, develop new programs and policies, and make financial decisions. Rebuilding Health Care Together: The Provincial Health Plan 2013-2018 is a made-in-New Brunswick guide to a sustainable health-care system. It supports the New Brunswick government’s strategic vision of a stronger economy and an enhanced quality of life while living within our means. | **Exchanging information:** Discusses the importance of cultural competency/ strengthening cultural sensitivity, specifically when working with First Nations patients (pg. 10). Discuss importance of multidisciplinary care to address patient health (pg. 11).  **Fostering healing relationship:** have a quote from First Nations patient, stating “Need for more dialogue/better relationship with health authorities – to know what needs are/what resources exist/what resources are missing*”* (pg. 23).  **Responding to emotions:** emphasizes putting clients at the centre of care through mental-health promotion, changes in service delivery in all sectors, the early identification of mental illness, effective intervention, and working to shift the stigma of mental illness (pg. 12). | ---- |
| Improving Health Together: A Policy Framework for Chronic Disease Prevention and  Management in Newfoundland and Labrador [57]  2011 | This document provides a comprehensive and coordinated approach that considers the common issues and needs of all those living with or at risk for chronic disease. This framework will guide our government over the next  several years as we work to manage and prevent chronic disease in Newfoundland and Labrador. | **Exchanging information: I**ndividuals must receive clear, consistent, and accurate information about their health and health care services to support them in leading healthier lives. The message needs to be presented in plain language at a level that can be understood by everyone (pg. 12). When individuals understand the reason for the approach to care and the outcomes that are expected, they are more likely to comply with medication regimes, have recommended tests and improve healthy behaviours to best manage their health (pg. 14). Also discusses linking patients to appropriate resources/services and discuss the importance of a multidisciplinary approach to care (pg. 13).  **Making decisions:** Discuss the importance of patients being more active in their own health (pg. 10). Discuss involvement of family (pg. 13).  **Enabling self-management:** Discuss improving chronic disease prevention and improving quality of life by patients managing their own health (pg. 1, 10). Discuss that it is the individual who decides, with the support of a health care provider, if and when they want to be more involved and are able to develop skills and confidence to manage their health (pg. 11). Discuss different resources that can help facilitate self-management support, including government and community-based group programs, self-help resources, web-based modules, video conferencing, and telehealth (pg. 11). The policy provides examples of initiatives that could support individuals in managing their health (pg. 11). | Mention the need to have targeted prevention efforts for at-risk populations including **women,** Aboriginal people, and individuals living with low income (pg. 8). |
| Report from the Canadian Chronic Disease Surveillance System: Hypertension in Canada [58]  2010 | To assess trends in prevalence of hypertension for administrative databases such as physician billing and hospitalization and resident registry databases (pg.2) | --- | --- |
| A Chronic Disease Prevention  and Management Framework  for New Brunswick [59]  2010 | This paper provides a succinct background and context to the need for a comprehensive New Brunswick chronic disease strategy. (p2) | **Exchanging information:**  Discusses importance of understanding patient needs and providing care that is culturally appropriate (pg. 16). All provider-patient interactions should be used as opportunities to inform patients about health promotion and disease prevention strategies. With the help of health care personnel, patients can engage in behaviours that prevent the onset of chronic conditions or delay complications (pg. 12). Provide patients with appropriate resources like community support (pg. 12). Specialists are integrated to provide support and expertise as required. This includes health promotion professionals who assist the general practitioner with community-based best practices (pg. 17).  **Making decisions:**  Health-care organizations creatively integrate explicit, proven guidelines into the day-to-day practice of the primary care providers in an accessible and easy-to-use manner. Guidelines and information are shared with patients to encourage their participation.  **Enabling self-management:**  Effective self-management is very different from telling patients what to do. Patients have a central role in determining their care – one that fosters a sense of responsibility for their health. Use effective self-management support strategies that include assessment, goal setting, and action planning, problem-solving and follow-up. Organize internal and community resources to provide ongoing self-management support to patients. Patients develop personal skills to maintain their health and wellness (pg. 16). Have modules on goal setting, action planning, managing challenges in chronic disease, etc. (pg. 16). Also discusses the need for providers to follow-up with patients and provide follow-up reminders (pg. 12). | --- |
