# Supplementary material for: Is patient-centred care for women a priority for policy-makers? Content analysis of government policies
Source: Health Res Policy Syst. 2020 Feb 18;18:23. doi: 10.1186/s12961-020-0533-z (PMC7029558; doi:10.1186/s12961-020-0533-z)
Supplement: Supplementary file 3 — Additional file 3. Women’s health domains addressed in included policies. Checklist of domains included in each policy. [file 12961_2020_533_MOESM3_ESM.docx]

**Additional File 3. Women’s health domains addressed in included policies**

**Depression (or mental health)**

| Policy | Women’s Health Domains | | | | | | Total (n) |
| --- | --- | --- | --- | --- | --- | --- | --- |
|  | Issues to consider for women | Social determinants of health | Issues specific to sub-groups | Distinguished care for women from men | Abuse or violence | Discrimination or stigma |  |
| Ontario Ministry of Health and Long- Term Care Estimates Briefing Book 2017-18, Canada 2018 [30] | --- | --- | X | --- | --- | --- | 1 |
| The Status of Women in Manitoba 2018 (Manitoba Ministry of Health, Canada, 2018) [31] | --- | --- | --- | --- | --- | --- | 0 |
| Mental Health and Addictions Realizing the Vision Better Mental Health Means Better Health, Canada 2017 [33] | --- | --- | --- | --- | --- | --- | 0 |
| Milestones on Our Journey Transforming Mental Health and Addictions in Nova Scotia A Provincial Model for Promoting Positive Mental Health, Care and Support (NS Health Authority, Canada, 2017) [35] | --- | --- | --- | --- | --- | --- | 0 |
| Transformational Roadmap Addiction & Mental Health Strategic Clinical Network (Alberta Health Services, Canada, 2017) [36] | --- | --- | --- | --- | --- | --- | 0 |
| Towards Recovery: The Mental Health and Addictions Action Plan for Newfoundland and Labrador (Government of Newfoundland and Labrador, Canada, 2017) [37] | --- | --- | --- | --- | --- | --- | 0 |
| Provincial Clinical and Preventative Services Planning for Manitoba: Doing things differently and better (Manitoba Ministry of Health, Canada, 2017)  [38] | --- | --- | --- | --- | --- | --- | 0 |
| Moving Forward Together, Prince Edward Island’s Mental Health and Addictions Strategy, covering the period 2016 to 2026 (PEI Mental Health Canada, 2016) [40] | --- | --- | --- | --- | --- | --- | 0 |
| Health Inequities in New Brunswick: A Report from the Office of the  Chief Medical Officer of Health, NB (New Brunswick Department of Health Canada, 2016) [41] | --- | --- | --- | X | --- | --- | 1 |
| Health PEI Annual Report 2016-2017 (Health Prince Edward Island, Canada, 2016) [42] | --- | --- | --- | --- | --- | --- | 0 |
| Provincial Advisory Council Addiction & Mental Health 2015-16 Annual Report (Alberta Health Services Canada, 2016) [44] | --- | --- | --- | --- | --- | --- | 0 |
| Enabling Effective, Quality Population and Patient-centred Care: A Provincial Strategy for Health Human Resources. (BC Ministry of Health, Canada, 2015) [45] | --- | --- | --- | --- | --- | --- | 0 |
| Primary and Community Care in BC: A Strategic Policy Framework  (BC Ministry of Health, Canada, 2015) [46] | X | --- | --- | --- | --- | --- | 1 |
| Progress Report The Action Plan for Mental Health in New Brunswick  2011–2018 (Government of New Brunswick, Canada, 2015) [47] | --- |  | --- | --- |  | --- | 0 |
| Healthy Environments, Healthy People 2015 Health Status of Manitobans Report (Government of Manitoba, Canada, 2015) [48] | X | X | --- | --- | --- | --- | 2 |
| New Brunswick’s Wellness Strategy 2014-2021 The heart of our future (New Brunswick Department of Health Canada, 2014) [50] | --- | --- | --- | --- | --- | --- | 0 |
| Healthy Minds, Healthy People A Ten-Year Plan to Address Mental Health  and Substance Use in British Columbia. Monitoring Progress: 2012 Annual Report (BC Ministry of Health, Canada, 2012) [54] | --- | --- | --- | --- | --- | --- | 0 |
| Provincial Mental Health and Addictions Advisory Council Activity Plan 2011-2014 (Government of Newfoundland and Labrador, Canada, 2011) [56] | --- | --- | --- | --- | --- | --- | 0 |
| Manitoba Women’s Health Strategy 2011(Ministry of Health Manitoba, Canada, 2011) [57] | --- | X | X | X | --- | --- | 3 |
| Report on the Health Status of Manitobans 2010 Priorities for Prevention:  Everyone, Every Place, Every Day (Manitoba Ministry of Health, Canada, 2011) [58] | --- | X | --- | --- | --- | --- | 1 |
| Total | 2 | 3 | 2 | 2 | 0 | 0 | --- |

**Cardiac rehabilitation (cardiovascular health)**

| Policy | Women’s Health Domains | | | | | | Total (n) |
| --- | --- | --- | --- | --- | --- | --- | --- |
|  | Issues to consider for women | Social determinants of health | Issues specific to sub-groups | Distinguished care for women from men | Abuse or violence | Discrimination or stigma |  |
| Report from the Canadian Chronic Disease Surveillance System: Heart Disease in Canada (Public Health Agency of Canada, Canada, 2018) [60] | --- | --- | --- | --- | --- | --- | 0 |
| Department of Health and Community Services’ 2017-2020 Strategic Plan (Government of Newfoundland and Labrador, Canada, 2017) [62] | --- | --- | --- | --- | --- | --- | 0 |
| Accreditation Report (Alberta Health Services, Canada, 2016) [63] | --- | --- | --- | --- | --- | --- | 0 |
| A Vision for Chronic Condition and Disease Prevention and Management (Alberta Health Services, Canada, 2016) [64] | --- | --- | --- | --- | --- | --- | 0 |
| Health Annual Report 2016–2017 (Province of New Brunswick, Canada, 2016) [65] | --- | --- | --- | --- | --- | --- | 0 |
| Setting Priorities for the B.C. Health System (British Columbia Ministry of Health, Canada, 2014) [66] | --- | --- | --- | --- | --- | --- | 0 |
| The Provincial Health Plan 2013-2018 (Province of New Brunswick, Canada, 2013) [67] | --- | --- | --- | --- | --- | --- | 0 |
| Improving Health Together: A Policy Framework for Chronic Disease Prevention and Management in Newfoundland and Labrador (Newfoundland Department of Health and Community Services, Canada, 2011) [68] | --- | X | --- | --- | --- | --- | 1 |
| Report from the Canadian Chronic Disease Surveillance System: Hypertension in Canada (Public Health Agency of Canada, Canada, 2010) [70] | --- | --- | --- | --- | --- | --- | 0 |
| A Chronic Disease Prevention and Management Framework for New Brunswick (Primary Health Care Branch Addiction, Mental Health and Primary Health Care Division Department of Health, Canada, 2010) [71] | --- | --- | --- | --- | --- | --- | 0 |
| Total | 0 | 1 | 0 | 0 | 0 | 0 | --- |
